# When Coronary Thrombosis Outpaces Technical Success

**DOI:** 10.1016/j.jaccas.2026.109096

**Published:** 2026-09-02

**Authors:** Rafael Alessandro Ferreira Gomes

**Affiliations:** Universidade de Pernambuco (UPE), Recife, Pernambuco, Brazil

**Keywords:** anticoagulation, antiplatelet, coronary artery bypass, hemostasis, postoperative, thrombosis

**Figure:**
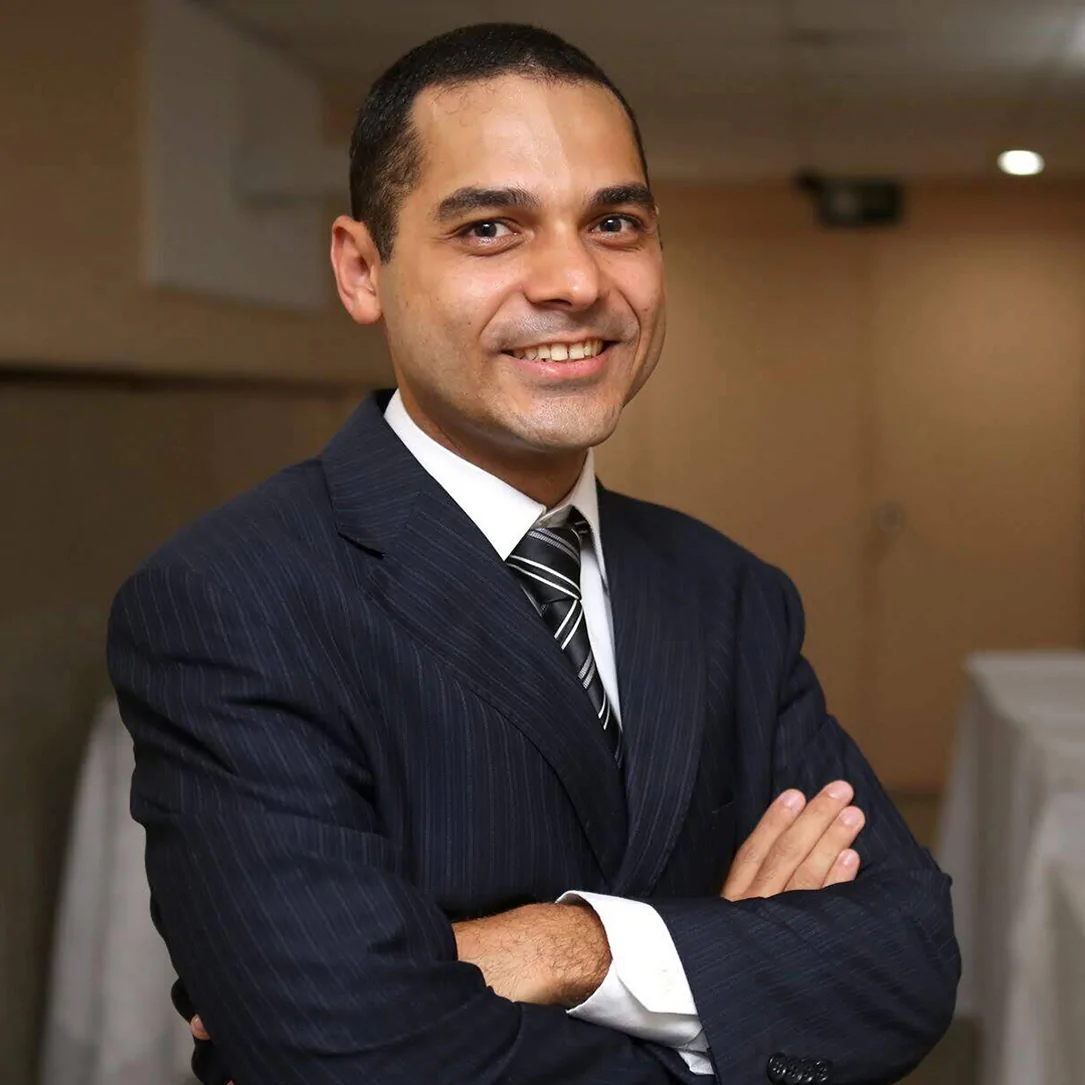

Coronary revascularization is usually judged by what the operator can see: a well-constructed graft, a patent anastomosis, a fully expanded stent, absence of dissection, and restoration of TIMI flow grade 3. These technical end points are essential. However, they do not always guarantee durable clinical success. The case reported by Kumari et al^1^ in this issue of *JACC: Case Reports*, illustrates a difficult but important concept: Sometimes coronary thrombosis progresses not because revascularization was technically unsuccessful, but because the patient's thrombotic biology is unusually hostile.

The clinical sequence is striking. A 68-year-old man with diabetes and multivessel coronary artery disease underwent elective coronary artery bypass grafting (CABG). In the early postoperative period, he developed lateral ST-segment elevation myocardial infarction, occlusion of saphenous vein grafts, and occlusion of the native obtuse marginal vessel distal to the graft anastomosis and then underwent percutaneous coronary intervention (PCI) of the native second obtuse marginal artery. Approximately 1 hour later, he developed acute stent thrombosis, followed by hemodynamic collapse requiring venoarterial extracorporeal membrane oxygenation. Further evaluation revealed a marked fall in platelet count, positive platelet factor 4/heparin immunoassays, persistently negative serotonin release assays, and later a *JAK2* V617F mutation consistent with essential thrombocythemia (ET).^1^

For the cardiologist, the first step in understanding this case is to resist a premature conclusion. Early graft occlusion after CABG should not automatically be interpreted as a technical surgical failure. It may result from several mechanisms, including conduit quality, anastomotic stenosis, target vessel runoff, competitive flow, low-flow states, perioperative inflammation, platelet activation, or systemic hypercoagulability. Similarly, acute stent thrombosis after PCI should first prompt a search for correctable mechanical causes, including stent underexpansion, edge dissection, malapposition, residual inflow or outflow disease, and high thrombus burden.

This is where the interventional assessment becomes crucial. In the catheterization laboratory, intravascular imaging helps distinguish a mechanical complication from a systemic thrombotic problem. In this case, intravascular ultrasound showed a well-expanded stent without dissection, making an obvious mechanical explanation less likely.^1^ This finding does not prove that systemic disease was the only cause of thrombosis, but it changes the clinical reasoning. Once major stent-related problems are excluded, the key question shifts from “what happened to the stent?” to “why is this patient thrombosing repeatedly?”

That shift is the central educational message of the case. Cardiologists are trained to think anatomically: which vessel is occluded, which graft failed, which segment needs treatment, and whether the stent result is optimal. This approach remains necessary. However, when thrombosis is early, recurrent, multifocal, and disproportionate to the technical findings, clinicians must move from lesion-based thinking to phenotype-based thinking. In other words, the question is no longer only where the thrombosis occurred, but what systemic condition is driving it.

Heparin-induced thrombocytopenia (HIT) is especially important in this context. Although its name emphasizes thrombocytopenia, HIT is primarily dangerous because it is a thrombotic disorder. Antibodies against platelet factor 4/heparin complexes activate platelets, generating a paradoxical state in which platelet counts fall while the risk of arterial and venous thrombosis rises.^2^ Therefore, after recent heparin exposure, the combination of a fall in platelet count >50%, new thrombosis, and a high 4T score should immediately raise concern for HIT.

The present case is also a reminder that HIT diagnosis may be clinically difficult. The patient had a high clinical probability and positive PF4/heparin immunoassays, but serotonin release assays remained negative. For this reason, the authors appropriately describe the diagnosis as presumptive HIT rather than definitive HIT.^1^ This distinction is important. A negative functional assay usually argues against HIT, but functional testing may be difficult to interpret in selected clinical scenarios, including treatment with potent P2Y12 inhibition.^3^^,^^4^ When antiplatelet washout is unsafe because of very recent stent thrombosis, clinicians may need to act before complete diagnostic certainty is available.

ET adds another layer to the case. ET is a chronic myeloproliferative neoplasm characterized by sustained thrombocytosis and increased risk of arterial and venous thrombosis.^5^^,^^6^ For cardiologists, the practical message is simple: Persistent thrombocytosis should not be dismissed as an incidental laboratory abnormality in a patient with arterial thrombosis. It may be the first visible clue to an occult clonal hematologic disorder. In this patient, chronic thrombocytosis, exclusion of reactive causes, and detection of *JAK2* V617F supported the diagnosis of ET.^1^

The *JAK2* V617F mutation is clinically relevant because it is associated with increased thrombotic risk in myeloproliferative neoplasms.^5^, ^6^, ^7^ Prior reports have also suggested that patients with *JAK2*-positive ET may have increased susceptibility to HIT and thrombosis, potentially through increased platelet activation and platelet factor 4 release.^8^^,^^9^ Thus, in this case, ET should not be viewed merely as a late diagnostic label. It may represent the biological substrate that made the patient unusually vulnerable to recurrent thrombosis after CABG, heparin exposure, and PCI.

The broader lesson is that graft occlusion, native vessel thrombosis, and acute stent thrombosis should not always be viewed as isolated events. They may be different vascular expressions of the same systemic thrombotic phenotype. The surgeon sees early graft failure. The interventional cardiologist sees acute stent thrombosis. The intensivist sees shock and multiorgan risk. The hematologist sees clonal thrombocytosis and possible immune-mediated platelet activation. The heart team must integrate all of these perspectives.

A practical framework may help (Figure 1). When coronary thrombosis recurs after CABG or PCI, clinicians should ask 5 questions in parallel:

1. Is there a technical or anatomical explanation, such as graft failure, anastomotic stenosis, poor target vessel runoff, stent underexpansion, dissection, or inflow/outflow disease?
2. Has intravascular imaging been used when appropriate to evaluate the stent result?
3. Is antiplatelet therapy adequate, absorbed, and biologically effective?
4. Does the timing of thrombocytopenia and heparin exposure suggest HIT?
5. Was there preexisting thrombocytosis or another clue to an occult myeloproliferative neoplasm?

**Figure 1:**
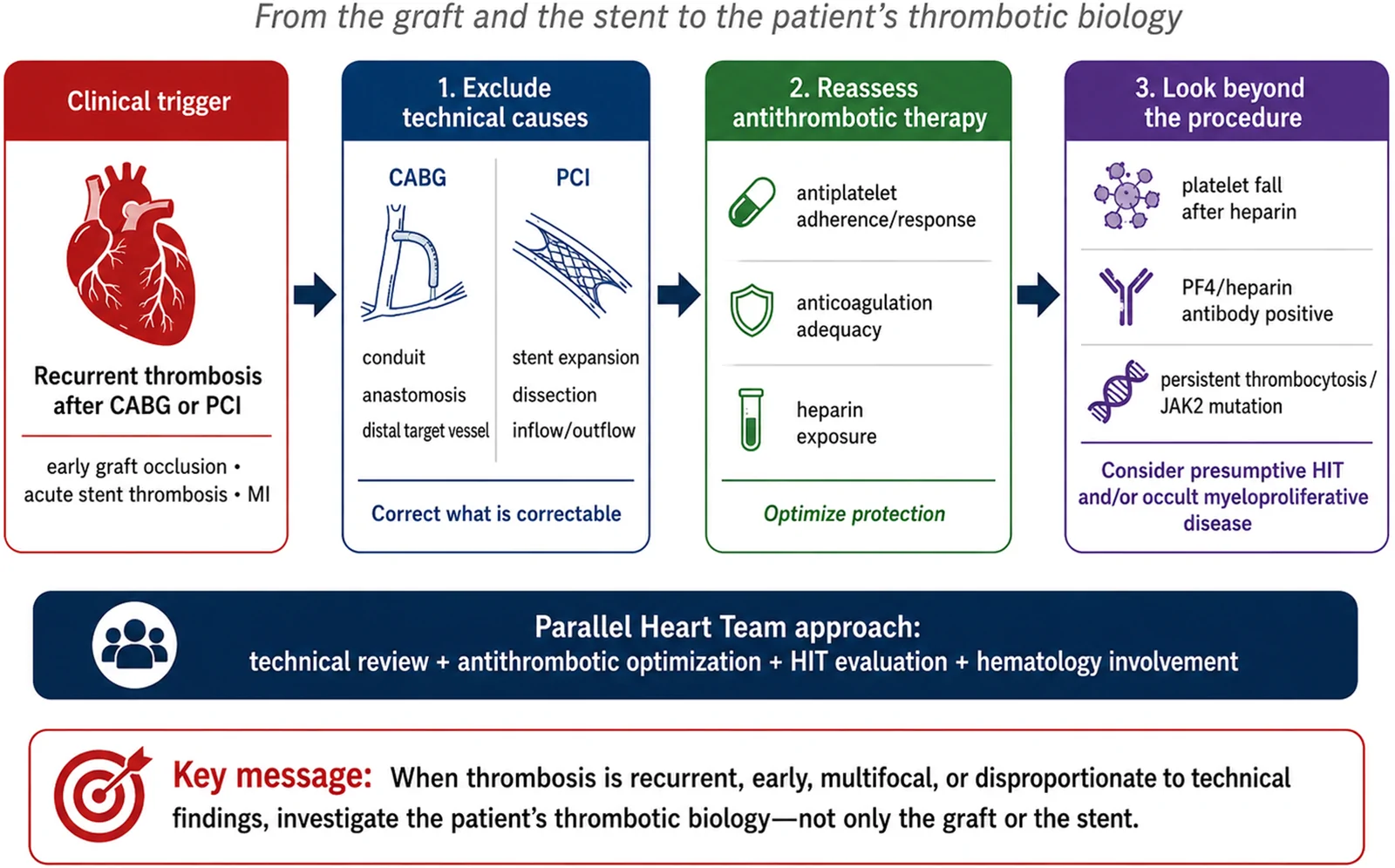
When Coronary Thrombosis Outpaces Technical Success Proposed approach to recurrent coronary thrombosis after coronary artery bypass grafting (CABG) or percutaneous coronary intervention (PCI). After excluding correctable technical causes, clinicians should reassess antithrombotic therapy and investigate underlying prothrombotic conditions, including heparin-induced thrombocytopenia (HIT) and occult myeloproliferative disease. The central message is that recurrent thrombosis may reflect the patient's thrombotic biology rather than procedural failure alone. MI = myocardial infarction; PF4 = platelet factor 4.

This case does not argue that every early graft occlusion or acute stent thrombosis reflects hematologic disease. Most do not. Rather, it teaches when to broaden the diagnostic lens. When coronary thrombosis is recurrent, early, multifocal, and disproportionate to the technical findings, the target of investigation should expand from the graft and the stent to the patient's thrombotic biology.

For the interventional cardiologist, the lesson is to interrogate the stent carefully, but not to stop at the stent. For the surgeon, early graft occlusion may sometimes be more than a conduit problem. For the heart team, the message is broader: Successful revascularization is not only an anatomical achievement, but also a biological negotiation. When coronary thrombosis outpaces technical success, the most important question may not be how to reopen the vessel, but why the vessel closed in the first place.

## Funding Support and Author Disclosures

The author has reported that they have no relationships relevant to the contents of this paper to disclose.
